# The transcription factor Zfp90 regulates the self-renewal and differentiation of hematopoietic stem cells

**DOI:** 10.1038/s41419-018-0721-8

**Published:** 2018-06-07

**Authors:** Ting Liu, Wei-xia Kong, Xiao-yi Tang, Man Xu, Qing-han Wang, Bin Zhang, Liang-ding Hu, Hu Chen

**Affiliations:** 1 Department of Hematopoietic Stem Cell Transplantation, Academy of Military Medical Sciences, Beijing, 100071 China; 2 Cell and Gene Therapy Center, Academy of Military Medical Sciences, Beijing, 100071 China

## Abstract

Hematopoietic stem cells (HSCs) can give rise to all blood cells that are essential to defend against pathogen invasion. The defective capability of HSC self-renewal is linked to many serious diseases, such as anemia. However, the potential mechanism regulating HSC self-renewal has not been thoroughly elucidated to date. In this study, we showed that Zfp90 was highly expressed in HSCs. Zfp90 deficiency in the hematopoietic system caused impaired HSPC pools and led to HSC dysfunction. We showed that Zfp90 deletion inhibited HSC proliferation, while HSC apoptosis was not affected. Regarding the mechanism of this effect on HSC proliferation, we found that Zfp90 interacted with Snf2l, a subunit of the NURF complex, to regulate *Hoxa9* expression. Ectopic expression of Hoxa9 rescued the HSC repopulation capacity in *Zfp90*-deficient mice, which indicates that Hoxa9 is the downstream effector of Zfp90. In summary, our findings identify Zfp90 as a key transcription factor in determining the fate of HSCs.

## Introduction

Hematopoietic stem cells (HSCs) generate all types of mature blood cells, which are essential for defense against pathogen infection. Although HSCs mostly exist in a quiescent state, they can quickly expand and differentiate in response to intrinsic or extrinsic cues, such as infection^1^. To maintain normal hematopoiesis, HSCs must maintain a balance between self-renewal and differentiation to preserve a constant hematopoietic stem progenitor cell (HSPC) pool and enough terminal hematopoietic cells. Hematopoiesis is elaborately regulated by signals and transcription factors^2,3^. Disorder of the regulation network often leads to the abnormal proliferation of HSCs and symmetric division. Dysregulation of particular transcription factors may lead to HSC exhaustion. Therefore, it is highly necessary to define the mechanism of transcriptional regulation in HSCs.

Zinc finger proteins (ZFP) are a diverse family of proteins, which conduct various biological functions. The ZFPs’ functional domains require at least one zinc ion to stabilize the integration of the protein itself^4^. Zinc finger-containing domains usually bind to DNA, RNA, proteins or small molecules to execute specific biological functions. The ZFP family can regulate gene expression in many tissues^5^. A previous study showed that Zfp90, a zinc finger protein, is involved in the regulation of cardiac development^6^. However, the role of Zfp90 in the hematopoietic system remains largely unknown. We found that Zfp90 is specifically highly expressed in HSCs compared with MPPs. Hence, we hypothesized that Zfp90 may play an essential role in HSC maintenance by regulating the expression of specific genes.

Chromatin modifiers have been shown to be important for gene expression during hematopoiesis. In most cases, chromatin is not accessible for transcription-factor binding and transcription initiation. Thus, chromatin remodeling is a prerequisite for gene expression^7^. Based on the common SWI/SNF-related catalytic ATPase subunit, chromatin remodeling complexes can be classified into four major subfamilies, including SWI/SNF, ISWI, CHD, and INO80^8^. Among these chromatin remodeling complexes, the nucleosome remodeling factor (NURF) complex, which contains three subunits of Bptf, Snf2l, and Rbbp4 in mammals, can use the energy from ATP hydrolysis to modify the chromatin structure to increase accessibility. Chromatin remodeling factors specifically associate with sequence-specific transcription factors to regulate gene expression^9,10^. A previous study has showed that the NURF complex participates in thymocyte maturation and HSC differentiation^11^. However, the function of the NURF complex in HSC maintenance has not been thoroughly elucidated to date. In this study, we showed that Zfp90 deletion causes rapid exhaustion of HSPC pools. Deletion of *Zfp90* using the CRSPR/Ca9 technology impairs the abilities of HSC self-renewal and repopulation. Zfp90 promotes HSC self-renewal via a Hoxa9-dependent fashion. Zfp90 associates with the NURF complex on the promoter of *Hoxa9* to initiate *Hoxa9* expression.

## Results

### Zfp90 is essential for the maintenance of HSPC pools

**Fig. 1:**
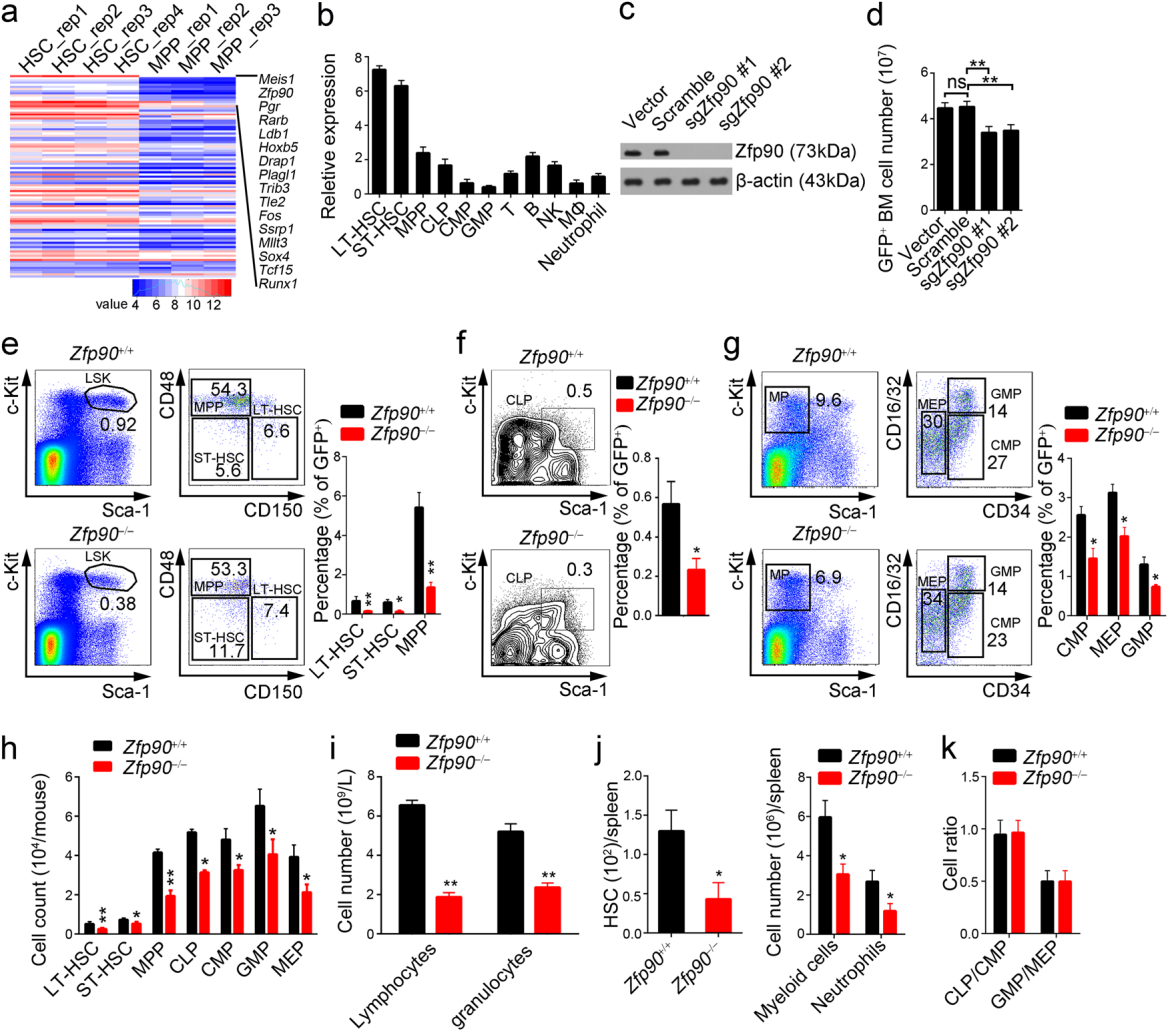
Zfp90 is essential for the maintenance of HSPC pools. **a** Expression profiles of transcription factors (TFs) in HSCs and MPPs were analyzed using R language and Bioconductor according to Jun Seita’s cohort (GSE34723). **b** Total RNA was extracted from representative hematopoietic populations. Expression levels of *Zfp90* were analyzed by real-time qPCR. Fold changes were normalized to endogenous *Actb*. **c** Expression levels of Zfp90 in indicative GFP^+^CD45.2^+^BM cells were evaluated by WB. β-actin was used to indicate the amount of loading proteins. **d** Analysis of whole CD45.2^+^GFP^+^ donor cell-derived bone marrow cells (BMC) in indicative chimera mice 16 weeks after transplantation. *n* = 6 for each group. **e** Analysis of long-term hematopoietic stem cells (LT-HSC), short-term hematopoietic stem cells (ST-HSC), and multipotential hemopoietic progenitors (MPP) in *Zfp90*^+/+^ and *Zfp90*^−/−^ mice 16 weeks after transplantation. *n* = 6 for each group. **f** Analysis of common lymphoid progenitors (CLP) in *Zfp90*^+/+^ and *Zfp90*^−/−^ mice 16 weeks after transplantation. CLP is gating on Lin^−^CD127^+^c-Kit^+^Sca1^+^. *n* = 6 for each group. **g** Analysis of common myeloid progenitors (CMP), granulocyte-macrophage progenitors (GMP) and megakaryocyte-erythroid progenitors (MEP) in *Zfp90*^+/+^ and *Zfp90*^−/−^ mice 16 weeks after transplantation. *n* = 6 for each group. **h** Numbers of representative hematopoietic progenitor cells in *Zfp90*^+/+^ and *Zfp90*^−/−^ mice were determined 16 weeks after transplantation. *n* = 6 for each group. **i** Peripheral blood cell counts were obtained from *Zfp90*-deleted and control mice 16 weeks after transplantation. Lymphocytes and granulocytes were analyzed. *n* = 6 for each group. **j** The numbers of HSCs, myeloid cells and neutrophils were calculated by FACS in the spleen from *Zfp90*^+/+^ and *Zfp90*^−/−^ mice 16 weeks after transplantation. **k** GMP/MEP ratio and the CMP/CLP ratio in *Zfp90*^−/−^ vs *Zfp90*^+/+^ mice were analyzed via FACS. *p<0.05 and **p<0.01 by two-tailed Student’s *t* test. All data presented are shown as the means ± SD collected from three independent experiments

HSCs are the source of all lineages of hematopoietic cells. Upon sensing differentiation signals, HSCs can differentiate toward multipotent progenitor cells (MPP) and MkE followed by common lymphoid progenitor cells (CLP) or common myeloid progenitor cells (CMP)^12,13^. To maintain the hematopoietic cell pool, HSCs need to maintain a balance between differentiation and self-renewal. Aberrant HSC self-renewal leads to impaired hematopoietic cell pools followed by serious nosohemia. To understand the regulatory mechanism of HSC self-renewal, we analyzed microarray data that was available online regarding HSCs and MPPs in Seita’s cohort (GSE34723) using R language and Bioconductor approaches^14,15^. Surprisingly, we found that many transcription factors were especially highly expressed in HSCs, among which *Zfp90* drew our attention (Fig. 1a and Supplemental Table 1). The *Zfp90* expression levels changed between HSCs and MPPs. To define the expression patterns of Zfp90, we purified mouse long-term hematopoietic stem cells (LT-HSC), short-term hematopoietic stem cells (ST-HSC), MPPs, CLP, CMP, granule-monocyte progenitors (GMP), CD3^+^ T cells, CD19^+^ B cells, macrophages and Gr1^+^CD11b^+^ neutrophils. Next, we analyzed the mRNA levels of *Zfp90* in these cells. We found that *Zfp90* was mainly expressed in isolated LT-HSCs and ST-HSCs (Fig. 1b).

To explore the role of Zfp90 in HSCs, we deleted Zfp90 in hematopoietic cells via the CRISPR/Cas9 technology using two different sgRNAs, as described before^16–18^. We infected WT bone marrow (BM) cells with lentivirus containing *Zfp90*-sgRNAs, scramble sgRNA or empty-vector control. As expected, Zfp90 was successfully deleted in GFP^+^ BM cells infected with either *Zfp90*-sgRNA, compared to that in the scramble sgRNA group or the empty-vector control group (Fig. 1c). Importantly, transfection with Zfp90-sgRNA did not affect the protein levels of the predicted off-target genes (Supplementary Figure 1a). To analyze the function of Zfp90 in vivo, we produced scramble-sgRNA-infected, empty-vector-infected and two Zfp90-sgRNA-infected chimeras by intravenously injecting 2 × 10^6^ GFP^+^CD45.2^+^ scramble-sgRNA, empty-vector control or zfp90-sgRNA BM cells into lethally irradiated CD45.1^+^ recipients. Sixteen weeks after transplantation, we found that the number of total BM cells was decreased in the chimeras derived from either Zfp90-sgRNA-infected BM cells compared to that in the scramble-sgRNA chimeras or empty-vector chimeras (Fig. 1d). Therefore, for the following analysis, we chose the scramble-sgRNA chimeras (hereinafter, we referred to these chimeras as *Zfp90*^+/+^) as control and Zfp90-sgRNA #1 for Zfp90 deletion (hereinafter, we referred to these chimeras as *Zfp90*^−/−^). Because mature BM cells were derived from HSPCs, we further examined the changes in hematopoietic progenitor cells in *Zfp90*-deleted mice. Interestingly, when Zfp90 was deleted, the percentages of LT-HSCs (GFP^+^Lin^−^Sca-1^+^c-Kit^+^CD150^+^CD48^−^), ST-HSCs (GFP^+^Lin^−^Sca-1^+^c-Kit^+^CD150^−^CD48^−^) and MPPs (GFP^+^Lin^−^Sca-1^+^c-Kit^+^CD150^−^CD48^+^) were decreased (Fig. 1e and supplementary Fig. 1b). Consistently, CLPs (GFP^+^Lin^−^IL7Rα^+^c-Kit^+^Sca-1^+^) (Fig. 1f and supplementary Fig. 1c), CMPs (GFP^+^Lin^−^c-Kit^+^Sca-1^−^CD34^+^CD16/32^−^), GMPs (GFP^+^Lin^−^c-Kit^+^Sca-1^−^CD34^+^CD16/32^+^) and MEPs (GFP^+^Lin^−^c-Kit^+^Sca-1^−^CD34^−^CD16/32^−^) (Fig. 1g and supplementary Fig. 1d) were also decreased. In addition, the total numbers of these progenitor cells were reduced (Fig. 1h). Notably, the percentages of GFP^+^CD45.2^+^ cells from the *Zfp90*^−/−^ mice or *Zfp90*^+/+^ control chimeras were similar in the femurs of recipient mice 18 h after transplantation (Supplementary Fig. 1e), indicating that the homing ability of *Zfp90*^−/−^ HSCs was not affected.

Next, we analyzed the hematopoietic cells in peripheral blood and found that the lymphocytes and granulocytes were decreased in the *Zfp90*-deleted mice compared with that in the *Zfp90*^+/+^ mice (Fig. 1i). Furthermore, we found that the HSCs in the spleen and splenocytes were also decreased in the *Zfp90*^−/−^ mice (Fig. 1j). However, we observed that the Zfp90 deletion did not affect the differentiation of HSCs toward either myeloid, erythroid or lymphoid lineages because the GMP/MEP and CMP/CLP ratios in the *Zfp90*^−/−^ and *Zfp90*^+/+^ mice were similar (Fig. 1k). In summary, Zfp90 deletion in mice leads to decreased HSPCs and impaired hematopoiesis.

### Zfp90 is indispensable for HSC self-renewal and repopulation capacity

**Fig. 2:**
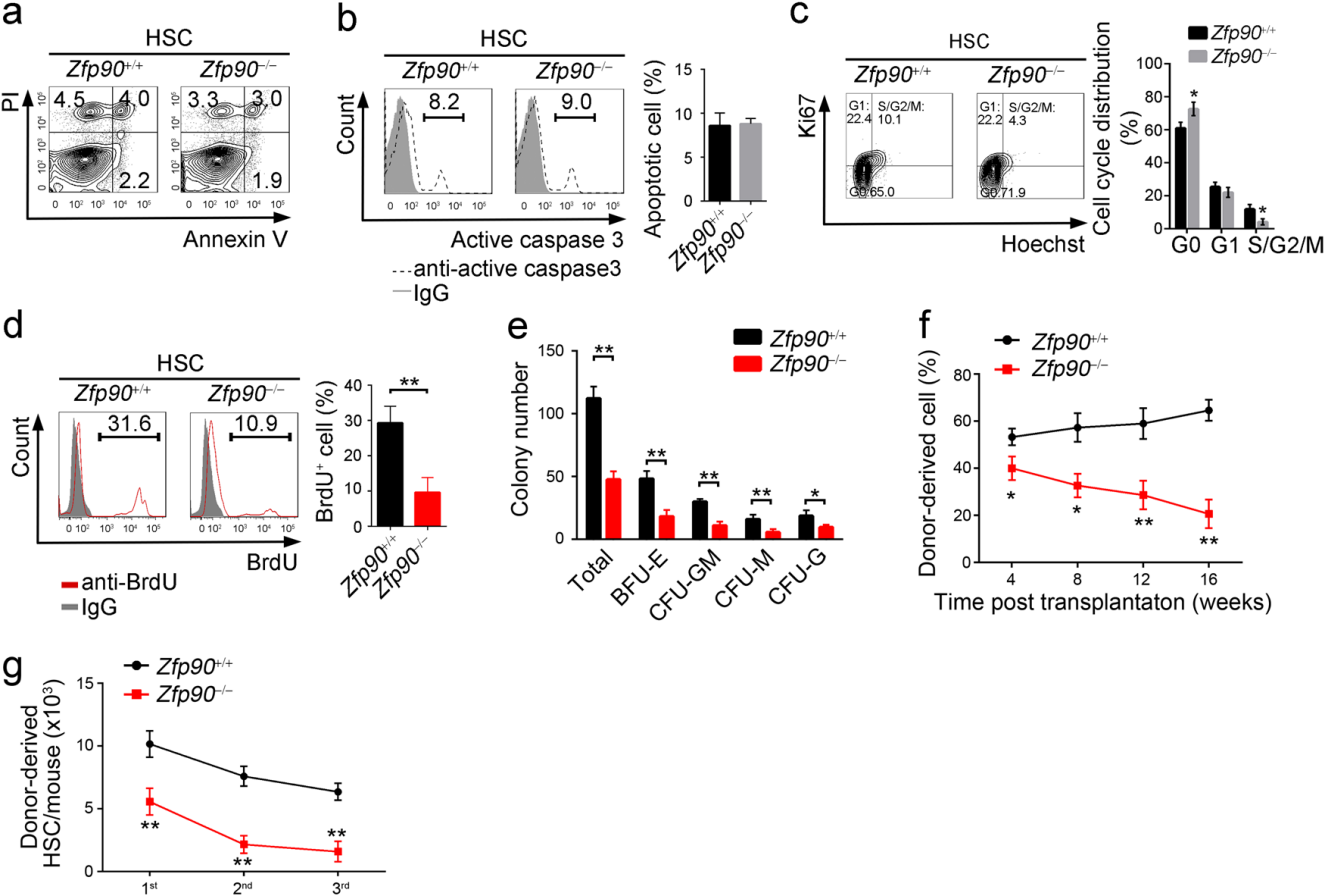
Zfp90 maintains HSC self-renewal ability and repopulation capacity. **a**, **b** Analysis of apoptotic HSCs (Lin^−^GFP^+^c-Kit^+^Sca-1^+^CD150^+^CD48^−^) in *Zfp90*^+/+^ and *Zfp90*^−/−^ mice via Annexin V/PI (**a**) and active caspase 3 (**b**) staining 16 weeks after transplantation. **c** Examination of HSC cell cycle via Ki67 staining in *Zfp90*^+/+^ and *Zfp90*^−/−^ mice 16 weeks after transplantation. Proliferative HSCs (Lin^−^GFP^+^c-Kit^+^Sca-1^+^CD150^+^CD48^−^) were defined as Ki67^+^Hoechst^+^ cells. *n* = 6 for each group. **d** *Zfp90*^+/+^ and *Zfp90*^−/−^ mice were i.p. injected with one dose (200 µg) of BrdU and fed continuously with water containing 800 µg/ml BrdU and 5% glucose for 3 days. Next, BrdU^+^ HSCs (Lin^−^GFP^+^c-Kit^+^Sca-1^+^CD150^+^CD48^−^) were analyzed by FACS. *n* = 6 for each group. **e** *Zfp90*^+/+^ and *Zfp90*^−/−^ HSCs were cultured in methocult medium for CFC formation assays. CFU-M, CFU-G, CFU-GM, and BFU-E colonies were counted. **f** 5 × 10^5^ *Zfp90*^+/+^ or *Zfp90*^−/−^ BM cells (CD45.2^+^) were transplanted along with 5 × 10^5^ CD45.1^+^ wild-type BM cells into lethally irradiated mouse. The mice were maintained on antibiotic drinking water for 14 days. Chimeras were periodically bled to assess the level of donor-derived blood cells at indicative time points. *n* = 6 for each group. **g** Enumeration of donor-derived HSCs per mouse 16 weeks after transplantation over serial transplantation. 1 × 10^3^ HSC (CD45.2^+^) from *Zfp90*^+/+^ or *Zfp90*^−/−^ chimeras were transplanted along with 5 × 10^5^ CD45.1^+^ BM cells into a lethally irradiated CD45^1^. recipient. *n* = 6 for each group. *p<0.05, **p<0.01 and ***p<0.001 by two-tailed Student’s *t* test. All data presented are shown as the means ± SD collected from three independent experiments

We showed above that the number of HSPCs was decreased in *Zfp90*^−/−^ mice. The decrease in cell number may be caused by cell death or proliferation. To explore the mechanism, we analyzed HSC apoptosis in *Zfp90*^−/−^ mice via Annexin V/PI and active caspase 3 staining. We found no obvious change in cell apoptosis after Zfp90 deletion (Fig. 2a,b). However, the proliferation rate of HSCs was reduced when Zfp90 was deleted. We found that the number of Ki67^+^ HSCs decreased in the *Zfp90*^−/−^ mice (Fig. 2c). The *Zfp90*^−/−^ HSCs incorporated much less BrdU than those from the *Zfp90*^+/+^ mice (Fig. 2d).

When the ability of HSC proliferation was impaired by Zfp90 deletion, we explored whether the differentiation and reconstitution capacities of HSCs were affected by Zfp90. First, we performed colony-forming cell (CFC) assays using MethoCult™ GF M3434 to define the potential of myeloid lineage colony formation. We found that *Zfp90*-deleted HSCs produced much fewer colonies in vitro, such as CFU-GM (Colony-forming unit-granulocyte-macrophage), CFU-M (Colony-forming unit-macrophage), BFU-E (Burst-forming unit-erythroid) and CFU-G (Colony-forming unit-granulocyte) colonies (Fig. 2e). Next, we conducted competitive bone marrow transplantation (BMT) assays to evaluate the capacity of HSC reconstitution. We transplanted a 1:1 mixture of CD45.2^+^ *Zfp90*^+/+^ or *Zfp90*^−/−^ and CD45.1^+^ WT BM cells into lethally irradiated CD45.1^+^ recipients. At indicative time points after the transplantation, we analyzed the percentages of CD45.2^+^ BM cells in the peripheral blood. Our data revealed that the *Zfp90*^−/−^ BM cells produced fewer blood cells (Fig. 2f). In addition, we performed serial BMT assays to analyze the role of Zfp90 on HSC maintenance. We transplanted 1 × 10^3^ *Zfp90*^+/+^ or *Zfp90*^−/−^ HSC (CD45.2^+^) from chimeras along with 5 × 10^5^ CD45.1^+^ WT helper BM cells into a lethally irradiated CD45.1^+^ recipient. After 16 weeks of the transplantation, we calculated the number of donor-derived HSCs in the chimeras. We found that the *Zfp90*^−/−^ HSCs decreased after serial transplantation (Fig. 2g). Taken together, these findings indicate that *Zfp90*-deleted HSCs showed reduced proliferation potential and impaired repopulation capacity. The cells rested on an abnormally quiescent status.

### Zfp90 deletion-mediated HSC loss is cell intrinsic

**Fig. 3:**
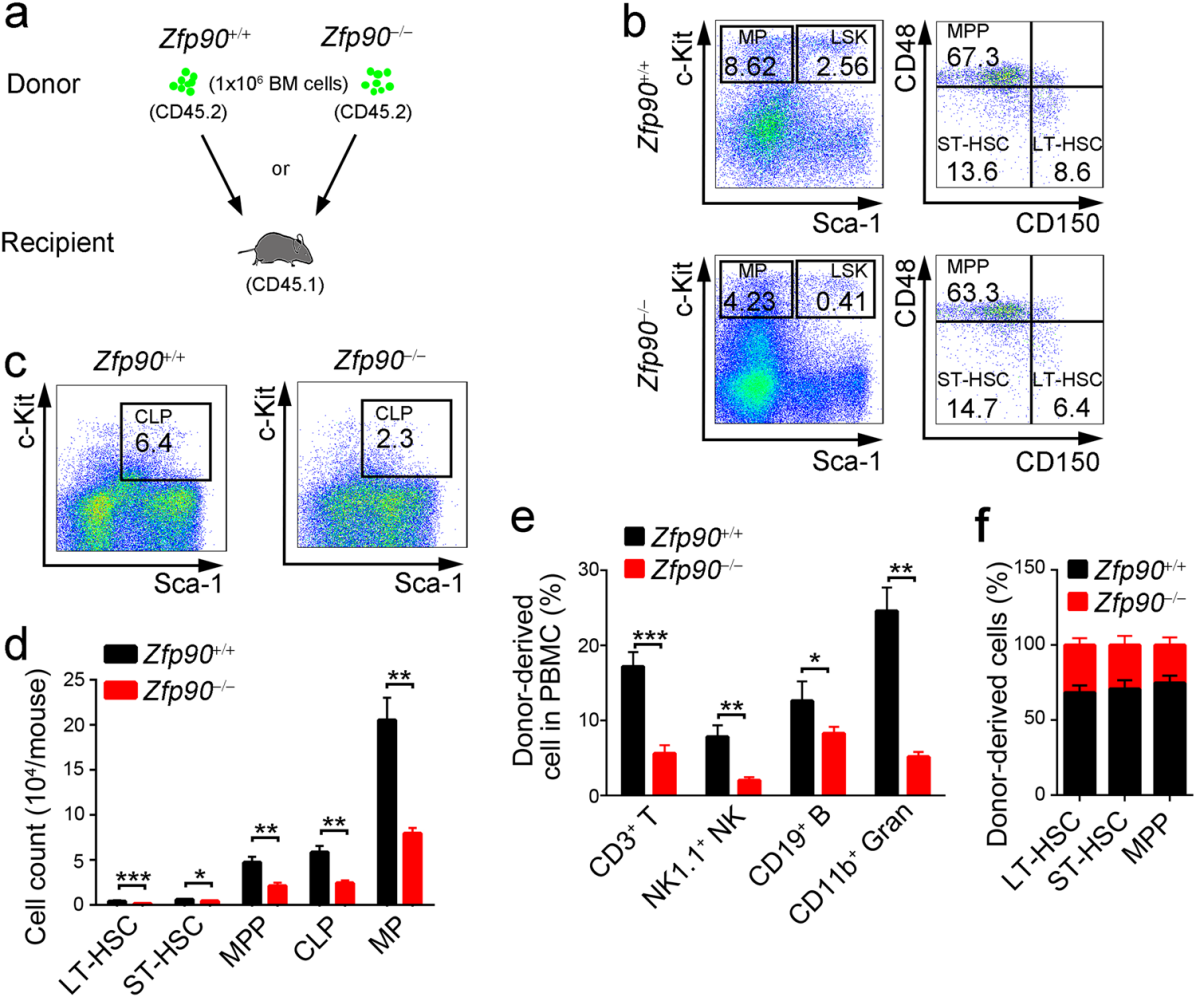
Zfp90-mediated HSC loss is cell intrinsic. **a** Schematic representation for BM transplantation assays. **b** Analysis of LT-HSCs, ST-HSCs, MPPs and myeloid progenitors (MP) in chimeras reconstituted with *Zfp90*^+/+^ or *Zfp90*^−/−^ BM cells. *n* = 6 for each group. **c** Analysis of CLPs in chimeras reconstituted with *Zfp90*^+/+^ or *Zfp90*^−/−^ BM cells. *n* = 6 for each group. **d** Enumeration of donor-derived HSCs, MPPs, CLPs and MPs in chimeras. *n* = 6 for each group. **e** Analysis of donor-derived CD45.2^+^ T, B, NK and Gran cells in the chimera blood. *n* = 6 for each group. **f** Flow cytometric analysis of the indicated cells from competitive BM chimera. Ratios of LT-HSCs, ST-HSCs and MPPs in chimeras were analyzed. *p<0.05, **p<0.01, and ***p<0.001 by two-tailed Student’s *t* test. All data presented are shown as the means±SD collected from three independent experiments

To determine whether Zfp90-deletion-mediated HSC abnormality is cell intrinsic or extrinsic, we performed BMT assays. We transplanted 1 × 10^6^ GFP^+^ *Zfp90*^+/+^ or *Zfp90*^−/−^ BM cells (CD45.2^+^) into lethally irradiated CD45.1^+^ recipients (Fig. 3a). After 16 weeks later, we analyzed the percentages of LT-HSCs, ST-HSCs, MPPs, CLPs, and MPs. We found that chimeras reconstituted with the *Zfp90*^−/−^ BM cells exhibit reduced HSPCs compared with those reconstituted with the *Zfp90*^+/+^ BM cells (Fig. 3b–d). Moreover, there was a decrease in the numbers of T cells, B cells, NK cells and granulocytes generated by *Zfp90*^−/−^ BM cells in blood (Fig. 3e). Furthermore, in the competitive BM transplantation assay, we found that Zfp90 deletion led to reduced percentages of LT-HSCs, ST-HSCs and MPPs in chimera BM (Fig. 3f). Collectively, Zfp90 acted as an intrinsic factor for HSC maintenance.

### Zfp90 associates with the NURF complex by interacting with Snf2l

**Fig. 4:**
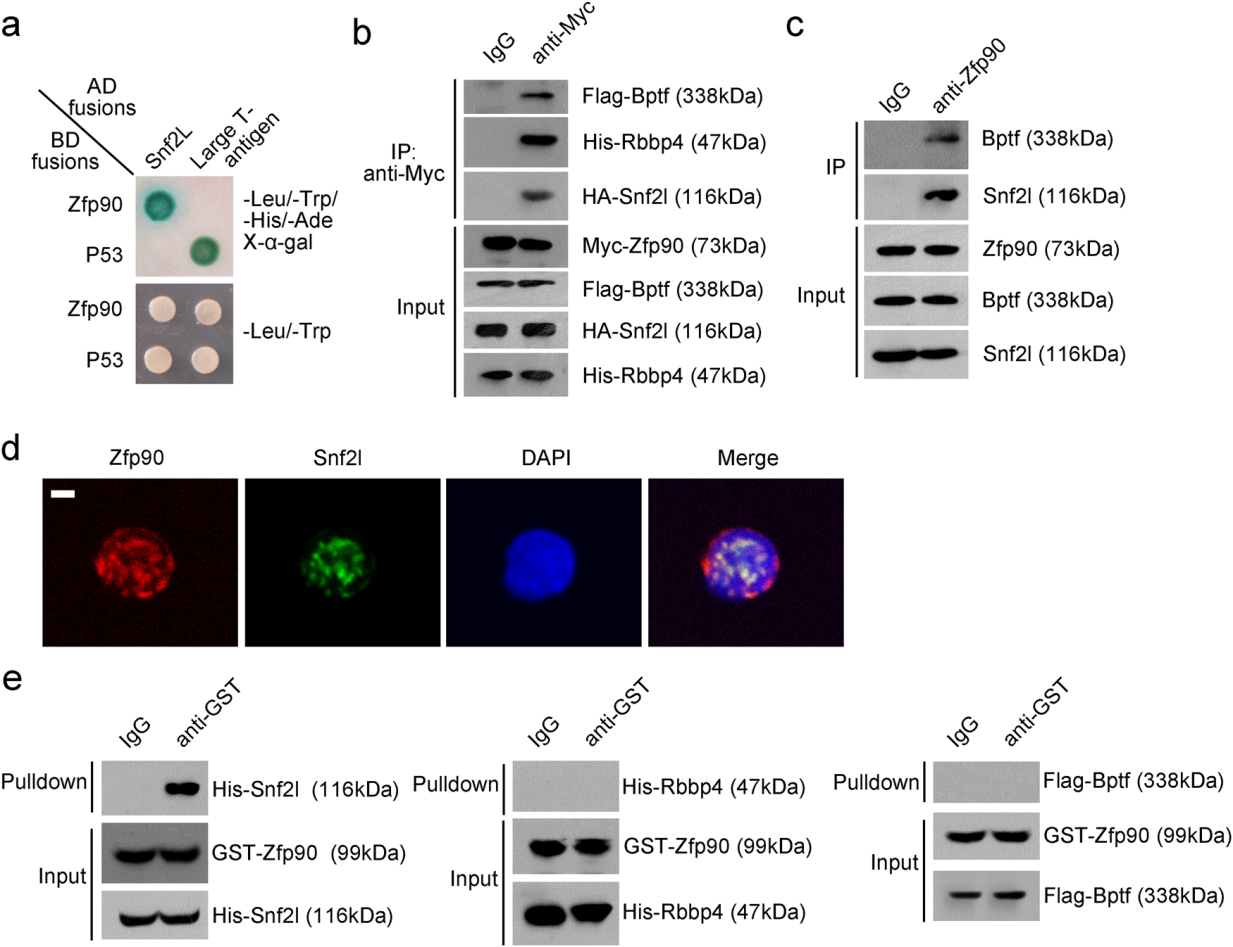
Zfp90 associates with the NURF complex by interacting with the Snf2l subunit. **a** Zfp90 interacts with Snf2l via yeast two-hybrid screen. Yeast strain AH109 was co-transfected with Gal4 DNA-binding domain (BD)-fused Zfp90 and Gal4 activating domain (AD)-fused Snf2l. Interaction of BD-p53 and AD-large T antigen was used as a positive control. **b** Interaction of Zfp90 with the NURF complex was confirmed by co-IP assay. Flag-Bptf, Myc-Zfp90, HA-Snf2l, and His-Rbbp4 were co-transfected into 293T cells for 36 h. β-actin was used to indicate the amount of loading proteins. **c** BM cell lysates were incubated with anti-Zfp90 for immunoprecipitation assay. β-actin was used to indicate the amount of loading proteins. **d** BM HSCs from wild-type mice were sorted, fixed and co-stained with the indicated antibodies. Zfp90, red; Snf2l, green; nucleus, blue. Scale bar=2 µm. **e** GST-tagged Zfp90 was incubated with His-tagged Snf2l, Rbbp4 or Flag tagged Bptf at 4 °C for 4 h, followed by incubation with GST beads and western blot (WB) analysis. β-actin was used to indicate the amount of loading proteins. All data are representative of three independent experiments

To explore the molecular mechanism through which Zfp90 regulated HSC maintenance, we performed a screen with mouse cDNA library using Zfp90 as a bait via the yeast two-hybrid approach. We identified Snf2l as a new potential candidate to interact with Zfp90 (Fig. 4a). Snf2l, also termed Smarca1, is an important component of the NURF complex that catalyzes nucleosome sliding and interacts with transcription factors to regulate gene expression. In mice, the NURF complex has three subunits of Bptf, Snf2l and Rbbp4. We confirmed the interaction of Zfp90 with the NURF complex via a co-immunoprecipitation (co-IP) assay (Fig. 4b). Our data showed that Myc-tagged Zfp90 enriched HA-Snf2l, His-Rbbp4, and Flag-Bptf (Fig. 4b). To examine the interaction in vivo, we conducted co-IP assays using BM cell lysates. We found that endogenous Zfp90 also interacted with Snf2l and Bptf (Fig. 4c). In addition, Zfp90 was co-localized with Snf2l in the nucleus of HSCs (Fig. 4d). To confirm whether the interaction of Zfp90 with NURF was direct or not, we purified the GST-Zfp90, His-Snf2l, His-Rbbp4, and Flag-Bptf proteins. Next, we performed pull-down assays and found that Zfp90 directly bound to Snf2l, but not to Bptf or Rbbp4 (Fig. 4e). In summary, we showed that Zfp90 associated with the NURF complex by directly binding to Snf2l.

### Zfp90 cooperates with the NURF complex to regulate Hoxa9 expression

**Fig. 5:**
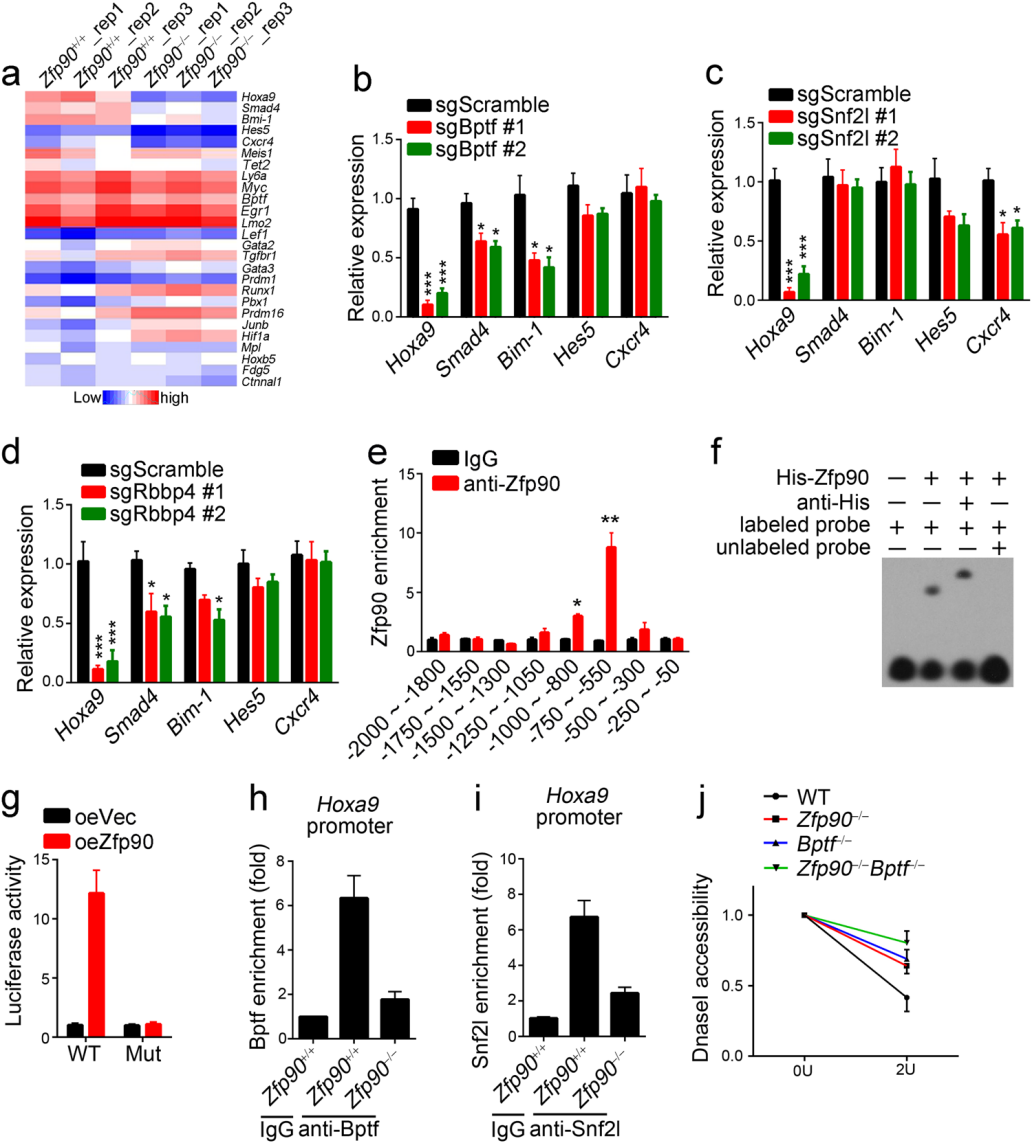
Zfp90 cooperates with the NURF complex to regulate *Hoxa9* expression. **a** Relative expression of representative HSC-proliferation-related genes. HSCs were isolated from *Zfp90*^+/+^ and *Zfp90*^−/−^ mice 16 weeks after transplantation. **b**–**d** Analysis of representative gene expression in *Bptf*-, *Snf2l*- or *Rbbp4*-deleted HSCs and WT control. Two sgRNAs were used for the deletion of Bptf, Snf2l or Rbbp4. **e** Analysis of Zfp90 enrichment on *Hoxa9* promoter in LSK (Lin^−^Sca-1^+^c-Kit^+^) cells via ChIP assays. **f** Analysis of the direct interaction of Zfp90 with *Hoxa9* promoter via EMSA assays. **g** Myc-Zfp90, pTK, and pGL3-*Hoxa9* WT (region: −2000 to 0) or Mutant (Mut region: deletion of −550 to −800) promoter were transfected into 293T cells for luciferase assay. **h**, **i** Analysis of Bptf or Snf2l enrichment on *Hoxa9* promoter in *Zfp90*^+/+^ or *Zfp90*^−/−^ LSKs. **j** Accessibility of *Hoxa9* promoter to DNase I in *Zfp90*^+/+^ or *Zfp90*^−/−^ LSKs was assessed. *p<0.05, **p<0.01, and ***p<0.001 by two-tailed Student’s *t* test. All data presented are shown as the means±SD collected from three independent experiments

Next, we sought to explore how Zfp90 cooperates with the NURF complex to regulate HSC maintenance. Previous studies have shown that many transcription factors (TFs) are involved in the regulation of HSC self-renewal, such as *Myc*, *Hoxa9*, *Gata2*, *Runx1*, *Gata3*, and *Lmo2*^19–24^. We explored whether Zfp90 regulates their expression in HSCs. Thus, we purified *Zfp90*^+/+^ and *Zfp90*^−/−^ HSCs and analyzed the expression levels of these TFs via RT-qPCR. Surprisingly, we found that the *Zfp90* deletion impaired *Hoxa9* expression (Fig. 5a). Considering that Zfp90 interacts with the NURF complex, we explored whether the NURF complex also regulates *Hoxa9* expression. We deleted *Bptf*, *Snf2l* or *Rbbp4* in HSCs via the CRISPR/Cas9 technology using two different sgRNAs and determined the *Hoxa9* expression levels. We validated the knockout of Bptf, Snf2l and Rbbp4 in BM cells via western blot analysis (Supplementary Fig. 1f) and found that the deletion of *Bptf*, *Snf2l* or *Rbbp4* by either sgRNA also led to decreased mRNA levels of *Hoxa9* (Fig. 5b-d). For further confirmation, we isolated LSKs (Lin^−^Sca-1^+^c-Kit^+^) that contain all HSCs to perform chromatin immunoprecipitation (ChIP) assays. We found that Zfp90 was enriched on the *Hoxa9* promoter (−750 to −550) (Fig. 5e). In addition, we confirmed their direct interaction through EMSA assays (Fig. 5f). Next, we conducted luciferase assays using the region (−2000 to 0 bp from transcription start site) of the *Hoxa9* promoter and found that Zfp90 overexpression promoted the luciferase activity, whereas deletion of the region (−800 to −550) in the luciferase reporter plasmid abrogated this trend (Fig. 5g).

Next, we evaluated how Zfp90 cooperated with the NURF complex to regulate *Hoxa9* expression. We performed ChIP assays with Bptf or Snf2l antibody and found that Zfp90 deletion impaired Bptf and Snf2l enrichment on the *Hoxa9* promoter (Fig. 5h, i). Moreover, Zfp90 or Bptf deletion decreased the chromatin accessibility of the *Hoxa9* promoter to DNase I digestion (Fig. 5j). Collectively, Zfp90 recruits the NURF complex to the *Hoxa9* promoter, and they cooperate to regulate *Hoxa9* expression.

### Hoxa9 rescues HSC self-renewal capacity in Zfp90 deficient mice

**Fig. 6:**
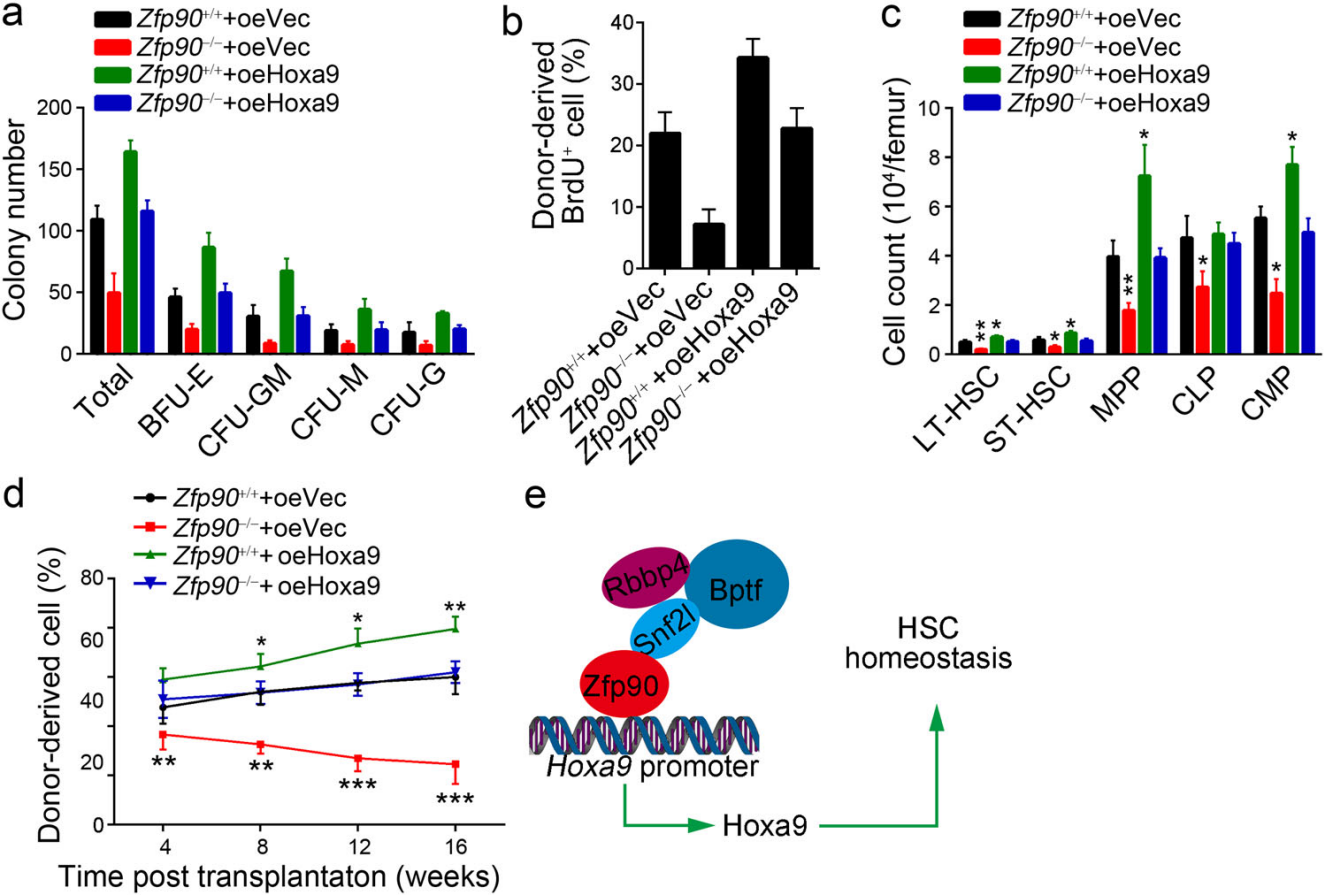
Hoxa9 rescues HSC self-renewal capacity in *Zfp90* deficient mice. **a** *Zfp90*^+/+^ or *Zfp90*^−/−^ HSCs were transfected with Hoxa9-overexpressing virus or vehicle and cultured in methocult medium for CFC formation assays. CFU-M, CFU-G, CFU-GM, and BFU-E colonies were counted. Results are shown as the means ± S.D. *n* = 6 for each group. **b** *Zfp90*^+/+^ or *Zfp90*^−/−^ BM cells were transfected with Hoxa9-overexpressing virus or vehicle and transplanted into lethally irradiated CD45.1^+^ mice. After 16 weeks, the chimeras were resolved with BrdU for 3 days, and donor-derived HSCs were analyzed by FACS on day 6. *n* = 6 for each group. **c** Numbers of LT-HSCs, ST-HSCs, MPPs, CLPs and CMPs in chimeras from (**b**). **d** 5 × 10^5^ *Zfp90*^+/+^ or *Zfp90*^−/−^ BM cells transfected with Hoxa9-overexpressing virus or vehicle were transplanted into lethally irradiated CD45.1^+^ mice supplemented with 5 × 10^5^ CD45.1^+^ helper cells for competitive BM transplantation assay. Donor-derived hematopoietic cells in the blood were analyzed via FACS. *n* = 6 for each group. **e** A schematic work model. Zfp90 regulates HSC homeostasis via recruiting the NURF complex to initiate *Hoxa9* expression. *p<0.05, **p<0.01, and ***p<0.001 by two-tailed Student’s *t* test. All data presented are shown as the means±SD collected from three independent experiments

To determine whether Hoxa9 is essential for HSC proliferation, we analyzed the effect of Hoxa9 ectopic expression via retrovirus in *Zfp90*^−/−^ HSCs. We found that overexpressing Hoxa9 restored the differentiation ability of the *Zfp90*^−/−^ HSCs in CFC assays (Fig. 6a). Moreover, we performed BMT assays. We transplanted 1 × 10^3^ *Zfp90*^−/−^ HSCs infected with Hoxa9-overexpressing virus into lethally irradiated recipients along with 5 × 10^5^ CD45.1^+^ WT BM cells. Sixteen weeks after transplantation, we conducted BrdU incorporation assays and found that Hoxa9 overexpression enhanced the BrdU incorporation by HSCs (Fig. 6b). In addition, ectopic Hoxa9 expression increased the number of LT-HSCs, ST-HSCs, MPPs, CLPs, and CMPs (Fig. 6c). As expected, Hoxa9 expression also restored the reconstitution capacity of the *Zfp90*^−/−^ HSCs (Fig. 6d). Notably, we showed that the overexpression of *Hoxa9* in *Zfp90*^+/+^ HSCs further promoted HSC expansion (Fig. 6a–d), which was consistent with a previous study^25^. Furthermore, we performed a BrdU incorporation assay using chimeras in the secondary BMT experiment and found that the overexpression of Hoxa9 also led to increased self-renewal of *Zfp90*-null HSCs (Supplementary Fig. 1g). In summary, *Hoxa9* is regulated by Zfp90 and plays a key role in Zfp90-mediated hematopoiesis (Fig. 6e).

## Discussion

In this study, we identified a novel transcription factor, Zfp90, involved in the maintenance of HSCs. We demonstrated that Zfp90 played a key role in maintaining HSC self-renewal and repopulation potential in vivo. Zfp90 intrinsically regulated HSC proliferation but not apoptosis. Regarding the mechanism, we identified that Zfp90 interacted with the NURF complex and synergistically regulated the chromatin accessibility of the *Hoxa9* promoter. Hoxa9 transcription activation was directly initiated by Zfp90 and the NURF complex. Hoxa9 acts as a downstream effector of Zfp90.

In adult mammals, hematopoiesis relied on a rare group of HSCs that rest in the bone marrow and possess the potential to self-renew and differentiate toward all lineage blood cells^26,27^. The self-renewal and differentiation of HSCs were elaborately regulated by internal and external signals. The cell cycle is essential for HSC proliferation and self-renewal^28,29^. However, HSCs need to maintain a balance between proliferation and quiescence to maintain normal hematopoiesis. For example, abnormal constitutive activation of Wnt signal leads to early HSC exhaustion^30^. Excessive activation of the Wnt/β-catenin signal promotes HSCs to enter into the cell cycle and lose the abilities of lineage differentiation and reconstitution^31,32^. In addition, previous reports showed that HSCs were activated during chronic infection through IFN-γ signaling^33^. Constitutive IFN-γ signaling also causes HSC exhaustion^33^. In contrast, loss of proliferation also leads to HSC dysfunction^34–36^. Many transcription factors have been demonstrated to be essential for HSC proliferation, such as *Hoxa9*^20^. *Hoxa9* deficiency results in impaired proliferation of HSCs. Here, we found that Zfp90 deletion led to the obvious inhibition of HSC proliferation and turnover rates. Zfp90 deletion reduced the expression levels of many transcription factors involved in the regulation of HSC proliferation including *Hoxa9*. However, the deletion of Zfp90 did not change HSC apoptosis. Thus, our study suggests that the decrease in HSC number in Zfp90-deleted mice is due to impaired proliferation.

*Hoxa9* is a member of the Hox gene family that contains a well conserved homeodomain and functions as transcription factors involved in embryonic development^37,38^. Previous research has showed that Hoxa9 plays an essential role in hematopoiesis^20^. *Hoxa9* is preferentially expressed in HSCs and other progenitors and is downregulated when HSCs differentiate^39^. Hoxa9 deletion in hematopoietic cells leads to a decrease in the number of CMPs^40,41^. In addition, another study showed that Hoxa9-deficient HSCs displayed impaired proliferation in vitro and did not differentiate into downstream progenitors, especially myeloid lineages^42^. Overexpressing Hoxa9 can rescue the proliferation and differentiation ability of HSCs. In vivo assays also showed that *Hoxa9* deletion weakened the HSC repopulating ability^43^. *Hoxa9*^−/−^ HSCs produced ~60% less myeloid cells compared to WT after bone marrow transplantation. However, Hoxa9-transgenic mice showed more number of HSCs and other progenitors in the BM^25^. Thus, Hoxa9 plays an indispensable role in HSC maintenance and differentiation, especially toward myeloid cells. Nevertheless, the regulation mechanism of *Hoxa9* expression remains unknown. In this study, our data show that the phenotype of Zfp90-deleted mice is similar to that of Hoxa9-mutant mice. Zfp90 acts as an upstream transcription factor to modulate *Hoxa9* expression. However, Zfp90 is highly expressed only at the HSC stage, unlike Hoxa9.

Epigenetic modifications including posttranslational modulation of histones, histone variant incorporation, DNA methylation and nucleosome remodeling activity regulate many biological processes, such as gene expression^44^. Exchange of histone variants and nucleosome remodeling rely on the existence of ATP-dependent chromatin remodeling complexes. Many chromatin remodeling complexes have been reported to regulate hematopoiesis. The SWI/SNF-like BAF complex is indispensable to regulate HSC survival^7^. Nucleosome remodeling deacetylase (NURD) is involved in HSC maintenance^45^. In this study, we found that Zfp90 binds to Snf2l, another subunit of the NURF complex, and recruits the NURF complex to the *Hoxa9* promoter. By promoting *Hoxa9* expression, Zfp90 and the NURF complex cooperate to control HSC proliferation and self-renewal. Our data reveal an additional function of the NURF complex in HSC maintenance.

## Materials and methods

### Cell culture

Human 293T cells (ATCC) were cultured in DMEM containing 10% FBS, 100 U/ml penicillin, 100 µg/ml streptomycin and 4 mM L-glutamine. Mouse multipotent HSC/MPP-like cell line EML cells (ATCC) were cultured in IMDM supplemented with 4 mM L-glutamine, 100 ng/ml mSCF, 20% FBS, 100 µg/ml streptomycin and 100 U/ml penicillin.

### Antibodies and reagents

Anti-CD127 (A7R34), anti-CD34 (RAM34), anti-c-Kit (2B8), anti-Sca-1 (D7), anti-CD16/32 (93), lineage cocktail (consisting of anti-CD3, anti-B220, anti-CD11b, anti-Ter119, and anti-Gr1, 88-7772), anti-CD150 (mShad150), anti-CD48 (HM48-1), anti-NK1.1 (PK136), anti-CD11b (M1/70), anti-CD3 (17A2), anti-CD19 (eBio1D3 (1D3)), anti-BrdU (BU20A) and anti-Ki67 (SolA15) were purchased from eBioscience. Anti-active caspase 3 (550821) was purchased from BD Bioscience. Antibodies against Myc (9E10) and GST (1-109) were purchased from Santa Cruz Biotechnology. Antibodies against Flag-tag (M1), β-actin (SP124), anti-Zfp90 (SAB2103688) and His-tag (6AT18) were obtained from Sigma-Aldrich. Anti-Snf2l (PA5-41440) and anti-Bptf (730026) were purchased from Invitrogen Antibodies. Antibodies conjugated with Alexa-488 (A11008) or Alexa-594 (A11012) was purchased from Molecular probes Inc. Hoechst 33342 (14533) were purchased from Sigma-Aldrich. Annexin V-FITC/PI apoptosis detection kit (BMS500FI) was purchased from eBioscience.

### Mouse strains, lentiviral production and bone marrow transplantation

Eight-week-old male C57BL/6 (CD45.2^+^) or SJL (CD45.1^+^) mice (~20 g) were purchased from Charles River and maintained under pathogen-free conditions. We replaced GFP with puromycin in lentiCRISPRv2 (Addgene, 52961) plasmid and cloned an sgRNA guide sequence targeting *Zfp90* into this vector. To produce lentivirus, lentiCRISPRv2-vector, lentiCRISPRv2-scramble or lentiCRISPRv2-sgRNA was co-transfected into 293T cells with packaging plasmids, pMD2.G (Addgene, 12259) and psPAX2 (Addgene, 12260), as described previously^46^. For *Zfp90* deletion in vivo, CD45.2^+^ WT BM cells were infected with 2 × 10^9^ infective units of lentiviruses in the presence of 2 µg/ml polybrene and were cultured in StemSpan SFEM (StemCell Technologies) supplemented with 50 ng/ml murine Thpo and 50 ng of murine Scf (both Peprotech) for 2 days. Next, 2 × 10^6^ GFP^+^ BM cells were isolated through FACS and intravenously injected into lethally irradiated CD45.1^+^ recipients.

For bone marrow transplantation, 1 × 10^6^ GFP^+^CD45.2^+^ *Zfp90*^+/+^ or *Zfp90*^−/−^ cells isolated from the *Zfp90*^+/+^ or *Zfp90*^−/−^ chimeras were collected via FACS and intravenously injected into lethally irradiated (10 Gy) CD45.1^+^ SJL recipients, as previously reported^47,48^.

For competitive transplantation, 5 × 10^5^ CD45.2^+^GFP^+^ *Zfp90*^+/+^ or *Zfp90*^−/−^ BM cells obtained as described above were intravenously injected into lethally irradiated CD45.1^+^ recipients together with 5 × 10^5^ CD45.1^+^ helper cells, as previously described^47^. At indicated time points post-transplantation, the percentages of donor-derived peripheral blood cells and other indicative cells were examined via FACS. Blood cells were obtained from the tail vein and were stained and analyzed via FACS.

Serial competitive transplantation was performed, as described previously^30^. In brief, for the first transplantation, 1 × 10^3^ GFP^+^ HSCs (CD45.2^+^) were generated as described above and injected into a lethally irradiated CD45.1^+^ recipient supplemented with 5 × 10^5^ fresh isolated CD45.1^+^ helper BM cells. For the second or third transplantation, 1 × 10^3^ HSCs (CD45.2^+^Lin^−^c-Kit^+^Sca1^+^CD150^+^) isolated from chimeras derived from the last transplantation were injected into a lethally irradiated CD45.1^+^ recipient supplemented with 5 × 10^5^ fresh isolated CD45.1^+^ helper BM cells. Sixteen weeks after transplantation, the number of HSCs was counted via FACS. All animal experiments were approved by the Institutional Animal Care and Use Committees at Academy of Military Medical Sciences. All animal experiments were conducted in accordance with the relevant guidelines and regulations of the Institutional Animal Care and Use Committees at Academy of Military Medical Sciences. Animals and protocols were approved by the Institutional Animal Care and Use Committees at Academy of Military Medical Sciences.

### Plasmids

Mouse Bptf coding sequence was cloned into a p3 × flag-CMV-9 expression vector. Mouse Rbbp4 full length was cloned into a pCDNA4-His expression vector. Mouse Snf2l was cloned into a pCDNA3-HA expression vector. Mouse Zfp90 was cloned into a pCDNA4-Myc expression vector. For recombinant protein purification, mouse Zfp90 was cloned into a pGEX-6P-1 vector, expressed in *E. coli* and purified with Glutathione Sepharose 4B beads, according to the manufacturer’s instruction. Mouse Snf2l was cloned into a Pet28a vector for *E.coli* recombinant expression and purification with Ni-NTA His-resins, according to the manufacturer’s instruction. Mouse Hoxa9 was cloned into a pMY-IRES-GFP vector for retrovirus production. Sg*Zfp90* (#1: 5′-ATTTCTTTCTCTGATATCCA-3′; #2: 5′-CTGCCCAGAGGAGCTTATAC-3′), sg*Bptf* (#1: 5′-CGCGAGCGCAGCCCCCCTAT-3′; #2: 5′-TATGAGGTGGTGCGGAACTT-3′), sg*Snf2l* (#1: 5′-TCTTTAAAGGTGGACGGCCC-3′; #2: 5′-CTTCTTGTTCTGTACGCCTG-3′) and sg*Rbbp4* (#1: 5′-TTCTTCCACTGCGTCGTCAA-3′; #2: 5′-GAGGTTTTGGTTCTGTCAGT-3′) were cloned into a lentiCRISPRv2-vector. Mouse *Hoxa9* promoter region was cloned into a pGL3 basic vector (Promega) for luciferase assays.

### Analysis of peripheral blood cells

The peripheral blood was collected from anaesthetized mice and was analyzed using an XFA6030 automated hemacytometer. Cell numbers and percentages of each population were counted.

### Flow cytometry

For BM cell analysis, the mice were sacrificed, and the BM cells were collected from the femurs in PBS containing 2% FBS. The cells were sifted through 70 µm cell strainers after removing red blood cells by suspending the cells in red cell lysis buffer. The cells were stained and analyzed via FACS. The staining strategy was performed as followed: LT-HSC (Lin^−^Sca-1^+^c-Kit^+^CD150^+^CD48^−^), ST-HSC (Lin^−^Sca-1^+^c-Kit^+^CD150^−^CD48^−^), MPP (Lin^−^Sca-1^+^c-Kit^+^Cd48^+^CD150^−^), CLP (Lin^−^CD127^+^Sca-1^+^c-Kit^+^), CMP (Lin^−^c-Kit^+^Sca-1^−^CD34^+^CD16/32^−^), GMP (Lin^−^c-Kit^+^Sca-1^−^CD34^+^CD16/32^+^), MEP (Lin^−^c-Kit^+^Sca-1^−^CD34^−^CD16/32^−^) and granulocyte (Gr1^+^CD11b^+^). Macrophages were isolated, as previously described^49^. For peripheral cell analysis, CD3^+^ T cells, CD19^+^ B cells, NK1.1^+^ NK cells and CD11b^+^ granulocytes in PBMCs were stained and evaluated via FACS. For cell cycle analysis, the HSCs were stained with indicated surface marker antibodies, followed by staining with Ki-67 and Hoechst 33342. For apoptosis analysis, the HSCs were stained with the indicated surface marker antibodies, followed by staining with PI/Annexin V or active caspase 3 antibodies. All data were analyzed using the FlowJo 7.6.1 software.

### Immunofluorescence assay

HSCs were isolated and placed on cationic slides and were fixed with 4% paraformaldehyde (PFA) for 20 min at room temperature. For nuclear protein staining, the HSCs were first resolved with 1% Triton X-100 permeabilization and 10% donkey serum blocking. The HSCs were incubated with indicative primary antibodies at 4℃ overnight, followed by incubation with the corresponding fluorescence-conjugated secondary antibodies for 1 h at room temperature. DAPI was used for nuclei staining. Images were obtained using an Olympus FV1000 laser scanning confocal microscope (Olympus, Japan). The ImageJ software was used for the quantitation of co-localization. For each experiment, at least 100 typical cells were observed.

### Immunoprecipitation assay

In total 293 T cells were co-transfected with the corresponding plasmids, maintained for 36 h and harvested. Next, the 293 T cells were lysed with ice-cold RIPA buffer (50 mM Tris–HCl, pH 7.4, 150 mM NaCl, 0.5% sodium desoxycholate, 0.1% SDS, 5 mM EDTA, 2 mM PMSF, 20 mg/ml aprotinin, 20 mg/ml leupeptin, 10 mg/ml pepstatin A, 150 mM benzamidine, and 1% Nonidet P-40) 4 °C for 2 h. The supernatant lysates were collected and incubated with indicated antibodies at 4 °C overnight. Finally, protein A/G agarose beads were added to the lysates, and immunoblotting was conducted.

### Real-time qPCR

Total RNAs from different populations of mouse hematopoietic cells were extracted using the RNA miniprep Kit (Tiangen, China) according to the manufacturer’s protocol. Next, 1 µg of total RNA per aliquot was used as a template for synthesizing cDNA with M-MLV reverse transcriptase (Promega, USA). For expression analysis of indicative genes, quantitative PCR analysis and data collection were performed using the ABI 7300 qPCR system. The qPCR primer sequences are available if requested.

### Chromatin immunoprecipitation (ChIP) assay

ChIP was performed according to a standard protocol (Upstate, USA). LSKs were purified, fixed in 1% formaldehyde for 10 min at 37 °C and lysed with ChIP SDS lysis buffer. DNA in the lysates were sheared into 200–500 bp by sonication. The lysates were incubated with 5 µg of the indicated antibody overnight at 4 °C, followed by immunoprecipitation with salmon sperm DNA/protein agarose beads. After washing, elution, cross-link reversal and purification, DNA from the ChIP sample was analyzed via qPCR.

### DNaseI accessibility assay

The BM cells were collected, and the LSKs were purified. The nuclei were isolated from the LSKs using the Nuclei isolating Kit (Sigma-Aldrich) according to the manufacturer’s protocol. The nuclei were resuspended in 150 µl of DNase I digestion buffer (1 mM EDTA, 0.1 mM EGTA, 5% sucrose, 1 mM MgCl_2_, and 0.5 mM CaCl_2_). Two equal aliquots of 75 µl of nuclei were treated with 0 or 2 units of DNase I (Sigma, USA) at 37 °C for 5 min. The reactions were stopped using 2 × DNase I stop buffer (20 mM Tris, pH 8.0, 4 mM EDTA, 2 mM EGTA). The DNA was extracted and analyzed using qPCR.

### Yeast two hybrid screening

Yeast two-hybrid screening was performed using the Matchmaker Gold Yeast Tow-Hybrid system (Clontech laboratories. Mountain View, USA) following the manufacturer’s instruction. In brief, mouse *Zfp90* was cloned into a pGBKT7 plasmid as BD-Zfp90 bait. Yeast AH109 cells were transfected with BD-Zfp90 and plasmids containing mouse spleen cDNA library (Clontech). The candidates were further identified via DNA sequencing.

### EMSA assay

EMSA experiments were conducted according to the manufacturer’s protocol using a Light Shift Chemiluminescent RNA EMSA Kit (Thermo Scientific). In brief, His-Zfp90 protein was incubated with a Biotin-labeled probe in the reaction system for 20 min at RT. The samples were analyzed in 4% polyacrylamide gel in 0.5 × TBE buffer. After being transferred on a nylon membrane (Amersham Biosciences), the labeled DNA was cross-linked by UV, checked with streptavidin-HRP conjugate and resolved using the detection substrate. The *Hoxa9* promoter sequence for EMSA was 5′-TCTTCTTCCTGCCGACAAGCGAGGGGGTGTGGATCCCGGGAGCTTCCCAGCCCCTCTCT-3′.

### BrdU incorporation

The mice were i.p. injected with one dose (200 µg) of BrdU and fed continuously with water containing 800 µg/ml BrdU and 5% glucose for 4 days. BrdU was detected via FACS using the BrdU labeling kit, as previously described^50^.

### Colony-forming assays

LSKs were isolated from wild-type C57BL/6 mice and infected with lentivirus containing *Zfp90*-sgRNA or a scramble sequence. One day later, GFP^+^ *Zfp90*^+/+^ or *Zfp90*^−/−^ HSCs (Lin^−^GFP^+^c-Kit^+^Sca1^+^CD150^+^) were sorted via FACS into a 96-well plate containing Methylcellulose Media (M3434; Stem cell technology). After incubation for 9–12 days, CFU-GM, CFU-M, BFU-E and CFU-G colonies were counted, as described previously^48^.

### Statistical analysis

An unpaired Student’s *t*-test was used for statistical analysis in this study. Statistical calculation was performed using Microsoft Excel or SPSS 13.

## Electronic supplementary material

Supplementary data
