# Supplementary material for: The transcription factor Zfp90 regulates the self-renewal and differentiation of hematopoietic stem cells
Source: Cell Death Dis. 2018 Jun 7;9(6):677. doi: 10.1038/s41419-018-0721-8 (PMC5992204; doi:10.1038/s41419-018-0721-8)
Supplement: Supplementary file 1 — Supplementary data [file 41419_2018_721_MOESM1_ESM.docx]

**
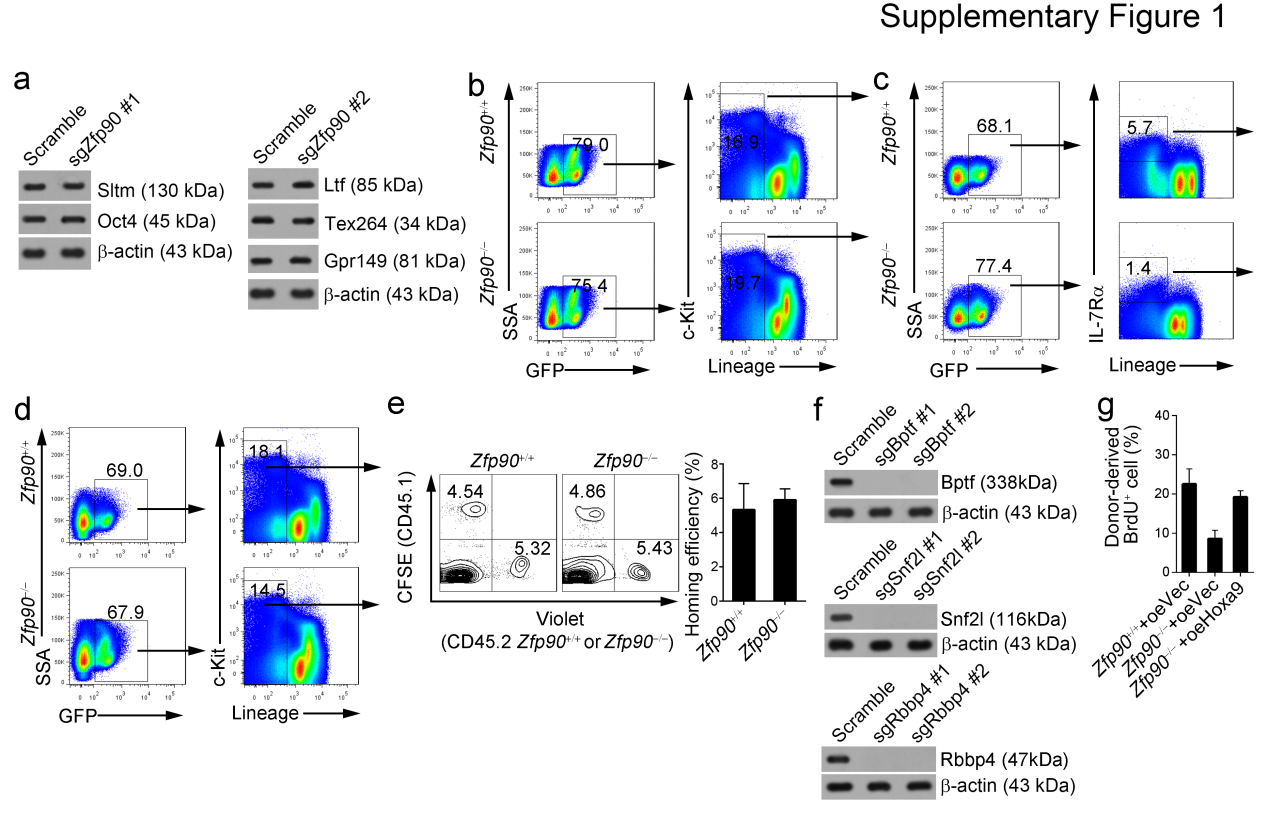
**

**Supplementary Figure 1: Zfp90 is essential for the maintenance of HSPC pools.** (**a**) Western blot was used to detect the protein levels of predicted off-target genes of sgZfp90 #1 and sgZfp90 #2 in BM cells. (**b**) FACS analysis for LT-HSC, ST-HSC and MPP in *Zfp90*^+/+^ and *Zfp90*^−/−^ mice. (**c**) FACS analysis for CLP in *Zfp90*^+/+^ and *Zfp90*^−/−^ mice. (**d**) FACS analysis for CMP, MEP and GMP in *Zfp90*^+/+^ and *Zfp90*^−/−^ mice. (**e**) Equal amounts of CD45.1 (CFSE labeled) and CD45.2 (Violet labeled) Lin^−^ BM cells were transplanted into lethally irradiated CD45.1 mice, followed by flow cytometry 18 h after transplantation. Homing efficiency was calculated as: absolute number of Violet^+^Lin^−^ cells 18 h after transplantation/absolute number of Violet^+^Lin^−^ cells transplanted. n = 6 per group. (**f**) Protein levels of Bptf, Snf2l and Rbbp4 in BM cells infected with sgRNAs were measured by western blot. (**g**)*Zfp90*^+/+^ or *Zfp90*^−/−^  BM cells infected with indicative plasmids were transplanted into lethally irradiated CD45.1^+^ mice for secondary BMT assays. 16 weeks later, chimeras were resolved with BrdU for 3 days and donor-derived HSCs were analyzed by FACS on day 6. n=6 for each group. **p*<0.05 and ***p*<0.01 by two-tailed Student’s *t* test. All data presented are shown as means ± SD collected from three independent experiments.

**Supplementary Table S1**

**Expression values (log_2_) of transcription factors analysed according to Seita’s cohort**

| **Symbol** | **HSC_rep1** | **HSC_rep2** | **HSC_rep3** | **HSC_rep4** | **MPP_rep1** | **MPP_rep2** | **MPP_rep3** |
| --- | --- | --- | --- | --- | --- | --- | --- |
| ***Meis1*** | 12.39991 | 12.41942 | 12.30989509 | 12.10751 | 8.979816 | 8.557246 | 8.511079 |
| ***Zfp90*** | 8.898619 | 8.753498 | 8.869842254 | 8.513288 | 5.572293 | 5.290829 | 5.041936 |
| ***Pgr*** | 8.213119 | 7.83666 | 7.911948753 | 8.208954 | 4.591171 | 4.507007 | 4.952692 |
| ***Rarb*** | 7.382424 | 7.425756 | 7.63778967 | 7.397232 | 3.967868 | 4.197191 | 4.438466 |
| ***Ldb1*** | 8.686191 | 8.555152 | 8.554010106 | 8.145403 | 6.001025 | 5.342771 | 4.890441 |
| ***Hoxb5*** | 7.298173 | 7.918028 | 8.448254007 | 8.739066 | 5.369941 | 5.251456 | 5.522953 |
| ***Drap1*** | 9.197815 | 9.019768 | 8.584259438 | 8.709068 | 6.36481 | 6.292644 | 5.597188 |
| ***Plagl1*** | 6.783438 | 6.185545 | 6.937233708 | 6.241408 | 3.823234 | 3.882188 | 4.157387 |
| ***Trib3*** | 9.116001 | 9.325669 | 8.423128647 | 9.10341 | 6.591717 | 6.332923 | 6.358291 |
| ***Tle2*** | 8.938937 | 9.304944 | 9.213570895 | 8.61829 | 6.624906 | 6.506533 | 6.506216 |
| ***Fos*** | 12.27772 | 13.73105 | 12.65615098 | 11.42268 | 11.36913 | 10.02258 | 7.558134 |
| ***Ssrp1*** | 11.45723 | 11.32783 | 11.26870186 | 10.96694 | 9.480634 | 8.747267 | 8.041447 |
| ***Mllt3*** | 12.38342 | 12.24795 | 12.56269427 | 12.27454 | 9.653473 | 9.954365 | 10.31776 |
| ***Sox4*** | 9.967556 | 10.20883 | 10.09633371 | 9.839831 | 8.073191 | 7.481383 | 7.351365 |
| ***Tcf15*** | 8.68257 | 8.746867 | 8.520219423 | 8.466026 | 6.090361 | 6.235448 | 6.520419 |
| ***Runx1*** | 10.5475 | 10.5572 | 10.07682721 | 9.917456 | 7.847597 | 7.895669 | 8.232364 |
| ***Hlf*** | 13.23654 | 13.39379 | 12.90157951 | 13.20439 | 10.26793 | 11.15154 | 11.15902 |
| ***Irf6*** | 10.13698 | 10.3627 | 10.31778172 | 10.42216 | 6.423736 | 8.356331 | 8.562372 |
| ***Tal1*** | 11.22201 | 11.19938 | 11.1885161 | 11.08182 | 8.179578 | 9.145702 | 9.230143 |
| ***Zfp82*** | 8.014365 | 7.654484 | 8.202066067 | 8.195847 | 5.997648 | 5.489136 | 5.853764 |
| ***Ppp5c*** | 8.576493 | 8.332745 | 7.710022439 | 8.28581 | 5.889552 | 6.207817 | 6.070258 |
| ***Abtb1*** | 9.167045 | 9.526421 | 8.961015656 | 9.233317 | 6.581202 | 7.478131 | 6.98842 |
| ***Nupr1*** | 7.223762 | 6.380748 | 6.806738434 | 6.518736 | 4.9281 | 4.204165 | 4.742059 |
| ***Taf1c*** | 9.513058 | 9.680328 | 9.527505611 | 9.680213 | 7.43217 | 7.603174 | 7.913146 |
| ***Nkx2-3*** | 8.102164 | 8.741669 | 8.265994138 | 7.966283 | 6.531334 | 6.388703 | 6.177726 |
| ***Vdr*** | 9.477104 | 9.42239 | 9.300779467 | 9.435948 | 7.082491 | 7.489104 | 7.821504 |
| ***E2f5*** | 6.702597 | 6.860858 | 7.051241925 | 7.47814 | 5.035425 | 5.342132 | 5.21908 |
| ***Epas1*** | 7.281842 | 6.917675 | 7.134493605 | 7.224214 | 5.018611 | 5.15152 | 5.743332 |
| ***Pbxip1*** | 8.523451 | 8.492952 | 8.385457202 | 8.525317 | 7.109892 | 6.624223 | 6.137377 |
| ***Zfp68*** | 10.41769 | 10.04891 | 10.14062569 | 9.895021 | 8.441426 | 8.305361 | 8.364879 |
| ***Dbp*** | 10.57078 | 10.8736 | 10.19734727 | 10.2642 | 8.274871 | 8.647877 | 9.258623 |
| ***Sqstm1*** | 9.697873 | 9.583934 | 9.122051651 | 9.353459 | 7.779823 | 7.664875 | 7.787655 |
| ***Cdk7*** | 9.228284 | 9.824076 | 9.673941954 | 9.821332 | 7.957792 | 8.134909 | 7.750114 |
| ***Trp53bp1*** | 8.34158 | 8.503859 | 8.593144266 | 7.869845 | 6.173489 | 7.034363 | 6.65861 |
| ***Aes*** | 7.876737 | 7.650989 | 6.915666628 | 7.456554 | 5.698526 | 5.996251 | 5.787381 |
| ***Pogk*** | 9.732448 | 9.426539 | 9.240901931 | 9.332359 | 7.804569 | 7.640463 | 7.906117 |
| ***Ppp1r16b*** | 8.279334 | 8.625299 | 8.783494993 | 8.653338 | 7.421914 | 6.412476 | 6.862777 |
| ***Ndn*** | 10.38254 | 10.23425 | 10.4792906 | 10.73611 | 8.801782 | 8.453646 | 9.178202 |
| ***Zfp111*** | 7.545947 | 7.307619 | 7.438249077 | 6.532332 | 5.208378 | 5.656511 | 5.973973 |
| ***Dedd2*** | 8.473686 | 8.502776 | 8.011609169 | 7.943978 | 6.214024 | 6.44898 | 7.129329 |
| ***Zfp40*** | 6.257039 | 5.525284 | 5.529791888 | 5.569534 | 4.331016 | 4.132458 | 3.980098 |
| ***Mta2*** | 9.096235 | 9.375951 | 9.395436842 | 9.193076 | 7.753807 | 7.872132 | 7.347318 |
| ***Hoxb6*** | 6.287802 | 6.352966 | 6.188876845 | 6.341999 | 4.45928 | 4.670981 | 4.940047 |
| ***Foxo1*** | 10.91138 | 11.17636 | 10.96618332 | 10.51013 | 8.526812 | 9.464999 | 9.777615 |
| ***Zfp54*** | 6.564316 | 6.080176 | 6.278338781 | 5.948465 | 4.958296 | 4.508422 | 4.506444 |
| ***Ezh1*** | 9.978787 | 10.49857 | 10.21577519 | 9.740239 | 8.707209 | 8.399617 | 8.599448 |
| ***Relb*** | 7.476369 | 6.917843 | 6.52129344 | 6.606465 | 5.459136 | 5.222934 | 5.524452 |
| ***Zfp354b*** | 6.57044 | 6.320654 | 5.597893881 | 5.651882 | 4.310091 | 4.539217 | 4.896202 |
| ***Phf10*** | 10.39279 | 10.21387 | 10.36954489 | 10.42695 | 7.9142 | 9.19055 | 9.245213 |
| ***Mkl1*** | 8.537586 | 8.694386 | 8.470587037 | 8.647993 | 7.202197 | 7.289947 | 6.975801 |
| ***Rnf14*** | 9.588591 | 9.60893 | 9.799293499 | 9.731903 | 7.18809 | 8.532517 | 8.697752 |
| ***Nfe2*** | 12.18209 | 12.3156 | 12.10751871 | 12.2524 | 10.32193 | 10.97441 | 11.07842 |
| ***Tle3*** | 9.013579 | 9.607195 | 9.123809816 | 9.348208 | 7.940045 | 7.914693 | 7.918515 |
| ***Ell2*** | 10.64998 | 10.62903 | 10.82556206 | 10.84931 | 9.322448 | 9.591068 | 9.23228 |
| ***Glis2*** | 8.818222 | 9.532081 | 8.937597612 | 9.157857 | 7.550257 | 7.734083 | 8.044999 |
| ***Hdac11*** | 9.676839 | 9.8135 | 9.145874257 | 9.025616 | 7.733766 | 8.010885 | 8.52186 |
| ***Zfp59*** | 7.740938 | 8.137635 | 7.612969178 | 7.521671 | 5.696366 | 6.557643 | 6.860957 |
| ***Spic*** | 5.932852 | 5.631038 | 4.229598582 | 4.973744 | 4.133804 | 3.886551 | 4.213217 |
| ***Nfatc2*** | 10.95178 | 11.2071 | 10.88464535 | 10.56981 | 9.362601 | 9.670084 | 9.990326 |
| ***Psmd10*** | 8.959351 | 8.889734 | 8.538639955 | 8.545199 | 7.297163 | 7.648387 | 7.614437 |
| ***Zfp1*** | 10.22686 | 10.09044 | 9.923221685 | 9.644696 | 8.890333 | 8.470197 | 8.948017 |
| ***Zfp292*** | 7.48217 | 7.517331 | 7.308654252 | 7.61617 | 6.258685 | 6.629012 | 5.915529 |
| ***Zfp467*** | 8.633316 | 9.04698 | 8.545259195 | 8.293829 | 6.670742 | 7.69858 | 7.811874 |
| ***Zfp287*** | 9.354766 | 9.543702 | 9.343559213 | 9.653553 | 7.951304 | 8.368491 | 8.554332 |
| ***Stat4*** | 10.92177 | 10.79694 | 10.59743124 | 10.51116 | 8.756053 | 9.744547 | 9.934508 |
| ***Irf1*** | 11.78184 | 11.81537 | 11.37889333 | 11.5543 | 10.19128 | 10.65975 | 10.82062 |
| ***Polr2e*** | 10.76252 | 10.41447 | 10.0569522 | 10.31159 | 9.088951 | 9.328762 | 9.59178 |
| ***Zfp93*** | 8.279892 | 8.043412 | 7.874915955 | 7.64469 | 6.941781 | 6.651642 | 7.141569 |
| ***Elk3*** | 9.698822 | 9.589078 | 9.240245567 | 9.171875 | 8.328952 | 8.347801 | 8.492034 |
| ***Olig1*** | 6.435893 | 3.918542 | 4.250704416 | 4.56937 | 4.056237 | 4.070581 | 4.294386 |
| ***Phf1*** | 6.923815 | 6.649742 | 7.146825845 | 6.591394 | 5.755403 | 6.125291 | 5.513009 |
| ***Tceal1*** | 9.348091 | 8.207864 | 8.183158405 | 8.808296 | 7.589463 | 7.514178 | 8.062986 |
| ***Pbx2*** | 8.134287 | 8.225158 | 7.92432063 | 7.996459 | 7.219151 | 7.040478 | 7.039991 |
| ***Zfp37*** | 6.444448 | 6.397756 | 6.318464073 | 5.402554 | 5.325057 | 5.012491 | 5.352151 |
| ***Taf1a*** | 8.300971 | 8.398375 | 8.460143862 | 7.8982 | 7.518102 | 7.430264 | 6.971674 |
| ***Zfp612*** | 5.901236 | 5.110968 | 5.673277183 | 5.574526 | 4.60419 | 4.760951 | 4.565366 |
| ***Phf13*** | 7.515642 | 7.50358 | 7.876107231 | 7.929994 | 5.715691 | 6.779727 | 7.375284 |
| ***Zfx*** | 5.973521 | 5.544604 | 5.765725459 | 5.326857 | 4.672286 | 4.840485 | 4.7686 |
| ***Khdrbs1*** | 10.82849 | 10.70031 | 10.26195093 | 10.80455 | 9.848947 | 9.761207 | 9.83681 |
| ***Creb3*** | 9.649081 | 9.769154 | 9.270572073 | 9.677094 | 8.780209 | 8.831196 | 9.019648 |
| ***Nfkbie*** | 10.40548 | 10.53509 | 9.967208739 | 10.05521 | 8.920236 | 9.717975 | 9.920422 |
